# The Role of Prediction in Learned Predictiveness

**DOI:** 10.1037/xan0000330

**Published:** 2022-07

**Authors:** Carla J. Eatherington, Mark Haselgrove

**Affiliations:** 1 Department of Human-Animal Interaction, Waltham Petcare Science Institute, Melton Mowbray, Leicestershire, United Kingdom; 2 School of Psychology, The University of Nottingham

**Keywords:** learning, attention, association, eye-tracking, learned predictiveness

## Abstract

Learning permits even relatively uninteresting stimuli to capture attention if they are established as predictors of important outcomes. Associative theories explain this “learned predictiveness” effect by positing that attention is a function of the relative strength of the association between stimuli and outcomes. In three experiments we show that this explanation is incomplete: learned overt visual-attention is not a function of the relative strength of the association between stimuli and an outcome. In three experiments, human participants were exposed to triplets of stimuli that comprised (a) a target (that defined correct responding), (b) a stimulus that was perfectly correlated with the presentation of the target, and (c) a stimulus that was uncorrelated with the presentation of the target. Participants’ knowledge of the associative relationship between the correlated or uncorrelated stimuli and the target was always good. However, eye-tracking revealed that an attentional bias toward the correlated stimulus only developed when it *and* target-relevant responding preceded the target stimulus. We propose a framework in which attentional changes are modulated during learning as a function the relative strength of the association between stimuli and the task-relevant response, rather than an association between stimuli and the task-relevant outcome.

The study of the relationship between learning and attention has a long history. Pavlov (1927) noted that a novel stimulus presented to an organism would elicit an “investigatory reflex” (p. 12), something that would today be referred to as an orienting response, and Lashley (1929) soon after, noted that only stimuli, or components of stimuli, that are organized by the principles of attention will become associated with one another. In the intervening 90 or so years there has been much discussion about the relationship between learning and attention (for reviews see Le Pelley, 2004; Le Pelley et al., 2016; Mackintosh, 1975; Pearce & Hall, 1980), but one feature of this relationship that has sustained interest is the extent to which a stimulus will come to attract attention if it is *predictive* of a subsequent event. In his influential theory of attention, for example, Mackintosh (1975) proposed that “subjects learn to attend to and ignore stimuli to the extent that those stimuli successfully predict the outcome of a trial,” and more recent theoretical analyses of learning and attention make similar claims (e.g., Esber & Haselgrove, 2011; George & Pearce, 2012; Le Pelley, 2004). This general principle has become known as the predictiveness principle, which refers to the idea that “*cues become more psychologically salient as a result of their predictiveness with respect to important outcomes; more attention will be allocated to predictive cues than to nonpredictive cues*” (Le Pelley et al., 2016, p. 8).

There seems to be good reason for advocating a general predictiveness-principle too. So called “learned predictiveness” tasks reliably show that stimuli that are good predictors of an outcome come to attract more overt visual attention than stimuli that are not. For example, in a study by Le Pelley et al. (2011) participants’ eye movements were recorded while they were presented with pairs of stimuli (nonsense words) on a computer screen, and on each trial asked to predict which of two different outcomes (sounds) would follow each pair. Across the experimental design, half of the stimuli were perfectly predictive of the identity of the outcome, while the remainder of the stimuli were irrelevant. The specifics of the design of the training can be seen in Table 1, where the nonsense words are represented as letters A to D, and V to Y. As can be seen, stimuli A and D were predictive of Outcome 1, and stimuli B and C were predictive of Outcome 2; thus, permitting the solution of this task. Stimuli V to Y, however, were presented on trials that terminated with Outcome 1 and Outcome 2. These stimuli were predictively redundant and consequently irrelevant to the solution of this task.[Table tbl1]

The results of Le Pelley et al.’s (2011) study revealed that, as a consequence of this training, participants’ visual dwell times were longer to the predictive stimuli than the irrelevant stimuli, a result that has been reproduced on a number of occasions using a variety of stimuli and tasks, in different laboratories (e.g., Alamia & Zénon, 2016; Aristizabal et al., 2016; Beesley et al., 2015; Griffiths & Mitchell, 2008; Haselgrove et al., 2016; Lochmann & Wills, 2003; Mitchell et al., 2012). It is also a result that concurs with studies of learning and attention in nonhuman animals (e.g., Haselgrove et al., 2010; Mackintosh & Little, 1969; Roberts et al., 1988).

Experimental results that conform to the predictiveness principle are often interpreted in terms of the framework provided by associative theories of learning. These theories stipulate that the attention paid to a stimulus can change according to some function of its associative strength, or the difference between its associative strength and the magnitude of the outcome: the so-called prediction error. To illustrate this, it is useful to consider the theory proposed by Mackintosh (1975), an influential model of learning in its own right, but one that has also been incorporated into more contemporary, and comprehensive treatments of learning (e.g., Le Pelley, 2004; Pearce & Mackintosh, 2010). According to Mackintosh, the change in the strength of the association between a stimulus (e.g., A) and an outcome (ΔV_A_) is determined by Equation 1:

$$\Delta V_{A}=\alpha \;\cdot \;\theta \;\cdot \;\left(\lambda \;–\;{\text{V}}_{A}\right)$$ 1

Here, the error term (λ – V_A_) is the discrepancy between the magnitude of the outcome (λ) and the current associative strength of stimulus A. θ is a learning rate parameter, determined by the properties of the outcome. Most important for the Mackintosh model, α is a variable stimulus-attention parameter that may increase or decrease after each trial. The rules proposed by Mackintosh for determining these increases and decreases in attention (Δα) are shown in Equations 2a and 2b, respectively:

$$\Delta \alpha_{A}>\;0\text{ if }\left|\;\lambda \;–\;{\text{V}}_{A}\right|\;<\;\left|\;\lambda \;–\;{\text{V}}_{r}\right|$$ 2a

$$\Delta \alpha_{A}<\;0\text{ if }\left|\;\lambda \;–\;{\text{V}}_{A}\right|\;\geq \;\left|\;\lambda \;–\;{\text{V}}_{r}\right|$$ 2b

where V_r_ is the sum of the associative strength of all stimuli present on that trial, minus V_A_ (i.e., it is the remainder). The size of the change in Δα_A_ is assumed to be proportional to the magnitude of the inequalities in Equations 2a or 2b. Using these equations, it can be seen how Mackintosh’s (1975) theory provides a mechanism for understanding the predictiveness principle. For example, the error terms of the predictive stimuli (A to D) in the study by Le Pelley et al. (2011), for example, will on each trial be less than the error terms of the irrelevant stimuli (V to Y). Consequently, it follows from Equation 2a that attention to predictive stimuli will increase, and from Equation 2b that attention to irrelevant stimuli will decrease.

One might wish to make the argument, however, that a sleight of hand is being performed when associative theories of attention, such as Mackintosh’s (1975) theory, are applied to our understanding of the predictiveness principle. Associative theories, rather paradoxically, generally say very little about how time is represented during learning (for discussions of this matter see Daw et al., 2006; Gallistel & Gibbon, 2000; Niv, 2009; Sutton & Barto, 2018). According to these theories, when events are paired, an opportunity is provided for an association to form between them. However, no information about the temporal relationship between the events themselves forms a part of the association—these models do not distinguish between events that are presented simultaneously and those that are presented sequentially. Consequently, the notion that a stimulus may be *predictive* of another event rather than merely *connected* to it, is something that is beyond the explanation of most accounts of associative learning. Consider Equations 1, 2a, and 2b, for example; these analyses of learning and attention make no explicit statement about the role of time, sequence or (crucially) prediction in learning. Instead, association and associative error are used to drive learning and attention; and yet conceptual understandings of learning and attention appeal to the role of prediction and prediction error to explain attentional phenomena such as the learned-predictiveness effect.

There has been rather little focus on the question of whether a sequential prediction is necessary for the establishment of a learned attentional bias, or whether mere association will suffice. In a visual search study reported by Beesley et al. (2018), participants were required to make a response about the orientation of a target stimulus that was presented simultaneously with an array of distractors. In one condition, a configuration of relevant distractors provided information about the location of the target. Despite the relationship between the configuration of distractors and the target, attention was not biased toward these configurations. At face value this might be taken to suggest that mere simultaneous association is not sufficient for the establishment of an attentional bias to a stimulus. However, what is unclear from this study is (a) whether participants had knowledge of the association between the configuration of distractors and the target and (b) if an attentional bias would have been established even if relevant distractors had been presented before the target (i.e., established as truly predictive).

The purpose of the experiments reported here was to uncouple association and prediction to determine their roles in learned changes in attention. To do this we investigated whether overt changes in attention were acquired to stimuli that were associatively correlated, or uncorrelated, with other events *in the absence of a sequential, predictive, relationship between them*. To anticipate our results, we observed that association between stimuli alone was insufficient to modulate an attentional bias. Instead, a learned attentional bias was only acquired to stimuli when predictive responding was necessitated by the task. We consider the role of the association between stimuli and task-relevant responding to explain these results.

## Experiment 1

Experiment 1 established a procedure in which stimuli were either correlated or uncorrelated with the presentation of a target stimulus, in the absence of any predictive relationship. The question of interest was whether, under these circumstances, visual dwell time would be longer to stimuli that were correlated with the target than stimuli that were uncorrelated with it. To achieve this, participants were trained with triplets of stimuli, each of which comprised a target stimulus (that participants were required to respond to), a correlated stimulus (that was presented with the target stimulus on 100% of the training trials), and an uncorrelated stimulus (that was presented with the target stimulus on only 50% of the training trials). The specifics of the design are shown in Table 2. It can be seen that during the training trials, stimuli U and V were perfectly correlated with the presentation of the targets Y and Z, respectively. However, stimuli W and X were uncorrelated with the target stimuli as they are presented equally frequently with Y and Z and provide no information about the identity of the target stimulus. Occasional test trials were presented that comprised only the correlated and uncorrelated stimuli.[Table tbl2]

The duration of participants eye gaze toward the correlated and uncorrelated stimuli was measured to determine whether participants acquired an attentional bias toward the correlated stimuli on the basis of their association with the target stimulus, relative to the uncorrelated stimulus. According to analyses that emphasize the importance of association in the acquisition of attention (e.g., Esber & Haselgrove, 2011; Le Pelley, 2004; Mackintosh, 1975) a stimulus that is correlated with the occurrence of the target will acquire more attention than a stimulus that is uncorrelated with the occurrence of the target. The same prediction does not necessarily hold if it is thought that prediction is an important determinant of learned changes in attention.

To determine participants’ knowledge about the relationship between the correlated and uncorrelated stimuli and the target, a final test was conducted in which participants rated the likelihood of the correlated and uncorrelated stimuli being paired with each target stimulus.

### Method

#### Participants

Eighteen participants (14 females; four males) were recruited from the University of Nottingham’s School of Psychology. Participants had a mean age of 19.6 years (*SD* = .61). All participants had normal or corrected-to-normal vision, and were excluded if they reported a history of visual disturbances triggered by flashing lights. Participants received course credit for their participation or a £3 inconvenience allowance. This experiment, as well as Experiments 2 and 3, received ethical approval from the institution’s local ethics committee.

#### Apparatus and Stimuli

The experiment was designed and run using experiment Builder Version 1.10.1630. An SR (Mississauga, Canada) Research Eyelink 1000 Plus eye-tracker sampled participant’s right eye at a rate of 1000 Hz. Gaze location was determined by monitoring the location of the pupil using an infrared camera mounted on the desk in front of the display monitor. Thresholds used to define fixations and saccades were: 15° displacement, 30°/s velocity, and 8,000°/s^2^ acceleration. Participants viewed stimuli binocularly from a distance of 67 cm, and head movements were minimized using a chin and forehead rest. Stimuli were presented upon a BenQ XL2420T LED monitor (33 × 57 cm) with a resolution set to 1,920 × 1,080 at 114 Hz. Stimuli consisted of the letters U, V, W, X, Y, and Z, presented in the color black, font size 60, Times New Roman. Stimuli were presented 5 cm apart from each other, at the apexes of a notional upright equilateral triangle the, center of which coincided with the center of the monitor screen. All stimuli were presented on a white background and subtended a visual angle of 25.4°. Regions of interest were set to 2.7 × 2.7 cm squares centered on each letter. Stimuli were counterbalanced as either the target, correlated, or uncorrelated stimuli.

#### Procedure

Eye movement data were recorded from each participant’s right eye. The eye-tracker was calibrated for each participant at the outset of the experimental session using a nine-point calibration, except for two participants who could only be calibrated using a 5-point procedure. A brief health questionnaire was administered to screen for exclusion criteria, written instructions were given, and informed consent was gained. At the start of each experimental session, the experimenter checked that both pupil and corneal reflections were present while participants read the on-screen instructions before performing a calibration. The written instructions for the calibration task were: “*Thank you for participating in this study. Your first task is to focus on the dot and follow it with your eyes. Press the space bar to begin the experiment.*” After performing the calibration, participants read further on-screen instructions: “*Each trial will begin with a fixation cross in the center of the screen. You need to look directly at the cross. Following this, your task is to indicate whether the letter (Y or Z) is present in the array. Try to remember which letters are paired together because at the end of the experiment you will have a memory test (Y = left, Z = right). Press any key to start the experiment.*” Nonitalicized text in brackets indicates example text that was counterbalanced for each participant.

Each trial began with the presentation of a 1 cm^2^ fixation cross located in the center of the screen for 1,000 ms. This was then replaced with either the triplets of stimuli (on training trials) or pairs of stimuli (test trials). The triplet stimuli remained on the screen until a response was made; the test trials stimuli remained on screen for 5,000 ms. Each trial was separated by an interstimulus interval of 1,000 ms, during which the screen was blank. Each of the four compounds of three stimuli shown in Table 2 was presented on 72 occasions across the experiment, providing 288 training trials in total. In addition, there were 24 test trials with the correlated and uncorrelated stimuli presented in the absence of the target stimulus (that may reasonably be expected to command most of the visual attention). No additional instructions were provided on these trials. Trial order was randomized over the whole experiment (312 trials in total). Stimulus position was varied randomly across the experiment to ensure that target, correlated, and uncorrelated stimuli were presented equally frequently in all three positions on both training and test trials.

After participants completed the experiment they were given two paper-based questionnaires each of which tested their understanding of the relationship between the correlated and uncorrelated stimuli with each target stimuli. At the top of the page was written, for example “*How likely was the letter ‘TARGET STIMULUS’ to be paired with the following letters?(please circle)*.” Presented underneath this question were the two correlated and two uncorrelated stimuli, next to each of which was a 10 point Likert scale that was anchored with the word “Unlikely” adjacent to the number 1, and the word “Likely” adjacent to the number 10. Upon completion of the questionnaires participants were thanked for their time and debriefed.

#### Transparency and Openness

For each experiment reported here, we detail processes for identifying any data to be excluded, any data exclusions, all manipulations, and all measures in the study. Data will be made available upon request to the corresponding author. The experiments were not preregistered.

### Results

Occasionally the eye-tracker lost track of pupil and corneal reflections, resulting in missing eye-movement data. Participants were excluded from further analysis if the percentage of missing data were greater than 20% (zero participants were excluded on this basis). Furthermore, if participants were excluded if they achieved less than 60% correct responses across all trial blocks (zero participants were excluded on this basis). For statistical analyses in this and subsequent experiments, Greenhouse-Geisser corrections were applied where sphericity was violated, however, degrees of freedom are rounded to the nearest whole number for the sake of clarity.

#### Behavioral Data

**Figure 1 fig1:**
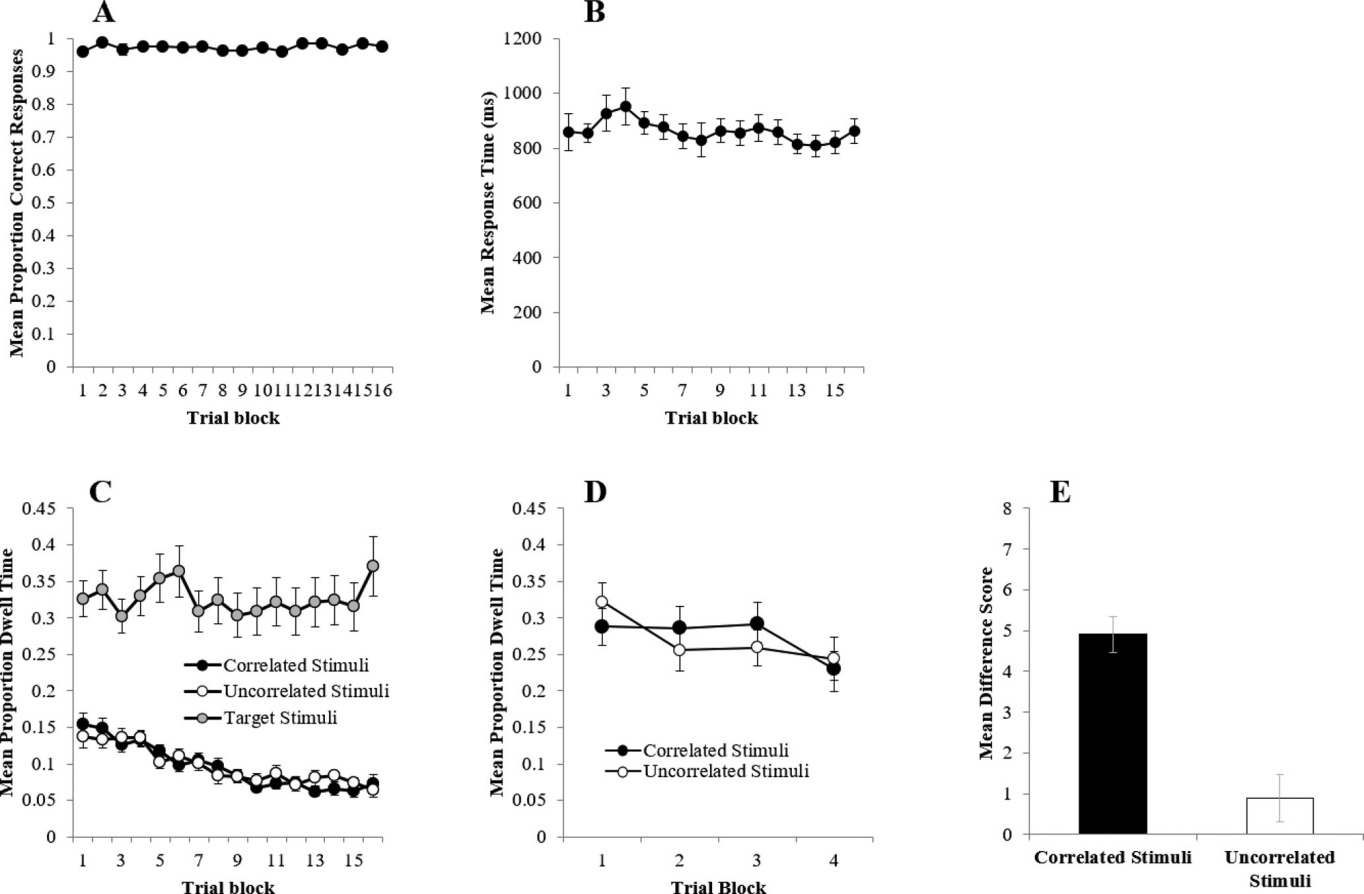
Results of Experiment 1 *Note.* (A) Mean proportion correct responses during the training trials; (B) mean response times during the training trials; (C) mean proportion dwell times to the correlated, uncorrelated, and target stimuli during training trials; (D) mean proportion dwell times to the correlated and uncorrelated stimuli during the test trials; and (E) mean difference scores to the correlated and uncorrelated stimuli during the final test questionnaire. Error bars represent 1 ± *SEM*.

Panel A of Figure 1 shows the mean proportion of correct responses over 16 blocks of 18 trials and reveals that participants performed the task accurately from trial Block 1, and by Block 16 had a mean accuracy of almost .96, which a one-sample *t* test revealed to be significantly above chance (.5), *t*(17) = 69.28, *p* < .001. A one-way repeated measures analysis of variance (ANOVA) of mean proportion correct with the factor of block (1–16) revealed a nonsignificant main effect, *F*(5, 87) = .73, η*_p_*^2^ = .04, *p* = .605, reflecting the lack of change over training. Similarly, the mean response time (RT), measured on correct and incorrect trials, from the termination of the fixation cross, varied very little across training (Figure 1, Panel B). A one-way repeated measures ANOVA of RT with the factor of block (1–16) also revealed a nonsignificant main effect, *F*(4, 60) = .66, η*_p_*^2^ = .04, *p* = .606.[Fig fig1]

#### Eye Gaze Analysis

Mean proportions of dwell time were calculated by dividing the total dwell time each participant spent within an ROI on each trial by the RT for that trial. These dwell times for the correlated, uncorrelated, and target stimuli during the training trials are shown in Panel C of Figure 1. This reveals that the target stimuli attracted the largest proportion of dwell time, and very little was directed either toward the correlated or the uncorrelated stimuli. A two-way repeated measures ANOVA of proportion of dwell time with the factors of stimulus type (target vs. correlated vs. uncorrelated) and trial block (1–16) revealed a significant main effect of stimulus type, *F*(1, 19) = 58.02, η*_p_*^2^ = .77, *p* < .001, trial block, *F*(4, 73) = 4.43, η*_p_*^2^ = .21, *p* = .002, and a significant interaction, *F*(22, 374) = 1.86, η*_p_*^2^ = .10, *p* = .050. Simple main effects analysis of this interaction revealed a significant difference between the proportion of dwell time directed toward target stimuli and the correlated or uncorrelated stimuli from block 1 onward, smallest, *F*(1, 19) = 26.03, η*_p_*^2^ = .51, *p* < .001, with the target stimuli always attracting a higher proportion of dwell time than the correlated and uncorrelated stimuli, which did not differ.

It was important to ascertain whether this nonsignificant difference between the correlated and uncorrelated stimuli supported the null hypothesis (there was no difference between the proportion of dwell time directed toward correlated and uncorrelated stimuli), or supported no conclusion at all. To decide between these two possibilities, a scaled JZS Bayes Factor was calculated according to the procedure described by Rouder et al. (2009) with a scale *r* = .707. The scaled JZS Bayes Factors for the difference between the correlated and uncorrelated stimuli was 4.08, which is in favor of the null.

One possible explanation for the absence of a difference between the proportion of dwell time directed toward the correlated and uncorrelated stimuli is that participants were directing so much attention toward the task-relevant target stimulus, that attention to the remaining stimuli on the screen was at floor levels. To examine this possibility, the 24 test trials where the target stimulus was absent were examined. Figure 1, Panel D shows that on the target-absent test trials, the mean proportion of dwell time directed toward correlated and uncorrelated stimuli was moderately longer than it was during the training trials that included the target (Figure 1, Panel C) but there was still no difference in dwell time between these stimuli. A two-way repeated measures ANOVA of proportion of dwell time with the factors of stimulus type (correlated vs. uncorrelated) and trial block (1–4) revealed a no effect of stimulus type, *F*(1, 17) = .04, η*_p_*^2^ = .002, *p* = .850, trial number, *F*(7, 112) = 1.76, η*_p_*^2^ = .09, *p* = .107, and no interaction between these factors, *F*(8, 133) = .73, η*_p_*^2^ = .04. The scaled JZS Bayes Factor for the difference between the correlated and uncorrelated stimuli was 4.04, which is in favor of the null.

#### Questionnaire Data

Participants were given a questionnaire that tested their understanding of the associative relationship between the correlated or uncorrelated stimuli with the target stimuli. A difference score was calculated to determine the specificity of the stimulus-target relationship. For the correlated stimulus, this was computed by subtracting the rating of the relationship between the correlated stimulus and the target stimulus it *was not paired with* from the rating of the relationship between the same correlated stimulus and the target stimulus that *it was paired with* (e.g., the rating of the relationship between U and Z was subtracted from the rating of the relationship between U and Y). For the uncorrelated stimulus, this was computed by subtracting the rating of the relationship between the uncorrelated stimulus and a target stimulus from the rating of the relationship between the same uncorrelated stimulus and the other target stimulus that it was paired with (e.g., the rating of the relationship between W and Y was subtracted from the rating of the relationship between W and Z): UY-UZ, VZ-VY, WY-WZ, XZ-XY. Panel E of Figure 1 reveals that the difference score was higher for correlated stimuli compared with uncorrelated stimuli, *t*(34) = 6.69, *p* < .001.

### Discussion

Participants received trials in which stimuli were either strongly positively correlated or entirely uncorrelated with the presentation of a task-relevant target. Participants engaged quickly and accurately with this task (Figure 1, Panels A and B), and showed clear knowledge about the associative relationship between the correlated stimuli and the target (Figure 1, Panel E). According to theories of learning that emphasize the role of changes in attention to stimuli as a consequence of association, these conditions should be sufficient for the acquisition of a bias in attention toward the correlated stimuli and away from the uncorrelated stimuli (e.g., Esber & Haselgrove, 2011; Le Pelley, 2004; Mackintosh, 1975). However, neither during training trials that included the target stimulus, nor during the test trials that included only the correlated and uncorrelated stimuli could we find any evidence of differences in the duration of visual dwell times, using either traditional frequentist statistics, nor with a calculation of Bayes factors (Figure 1, Panels C and D). These results imply that mere association, or differences in associative error, alone, are not sufficient for the establishment of differences in learned variations in attention.

## Experiment 2

In Experiment 1, we arranged for one set of visual stimuli to be positively correlated with a target stimulus while another set of stimuli were not. Despite the presence of appropriate associative knowledge about the relationship between the correlated or uncorrelated stimuli and the target, there was no difference in the extent to which participants’ overt visual attention was directed toward these stimuli. As we have noted, these results imply that mere association with a task relevant stimulus *alone* is not sufficient for the acquisition of an attentional bias to other stimuli (e.g., Esber & Haselgrove, 2011; Le Pelley, 2004; Mackintosh, 1975). Instead, these results are consistent with the idea that a predictive relationship must be arranged between stimuli for associations to be translated into changes in attention—an idea that is implied by the predictiveness principle (Le Pelley et al., 2016). However, while real and nonsense words are regularly used as visual stimuli in studies of the relationship between learning and attention (e.g., Le Pelley et al., 2011) it is relatively rare for single letters to be used in the same role. It is possible, then, that the stimuli used in Experiment 1 were, in and of themselves, unable to support learned variations in attention, irrespective of whether a predictive relationship was arranged between them or otherwise. The aim of Experiment 2, therefore, was to use single-letter stimuli as in Experiment 1, with the same degrees of correlation or noncorrelation between themselves and the target but under circumstances in which *a predictive relationship* is also embedded into the trial structure to explore whether, now, a bias in learned attention would develop. To do this, three groups of participants were included in Experiment 2. First, we included a group (group Simultaneous) that closely replicated the conditions of Experiment 1 to examine whether the outcome of this study would reproduce. A second group (group Serial-Target) received identical instructions to participants in group Simultaneous, but for this group the correlated and uncorrelated stimuli were presented simultaneously, as a pair, and then followed immediately afterward with the target stimulus—thus, reproducing the temporal arrangement of cues and outcomes more commonly used in studies of learned predictiveness. Finally, a third group (group Serial-Stimuli) received an identical trial structure to group Serial-Target; however, for this group the instructions were subtly changed. For group Serial-Stimuli, participants were again informed that they could respond when the target appeared, however, they were also informed that they could make a response earlier—before the presentation of the target during the preceding stimuli—if they thought they were able to predict its arrival.^1^ The purpose of including group Serial-Target and group Serial-Stimuli was to explore whether it was sufficient for just a predictive *temporal* relationship to be established between the correlated and target stimuli for an attentional bias to develop toward these stimuli; or, instead, whether in addition it was necessary for a predictive *response* to be made about the identity of a forthcoming, future event.

On the basis of the results of Experiment 1, we expected to see no difference in dwell times toward the correlated and uncorrelated stimuli in group Simultaneous. What remained to be determined, however, was whether the two groups that had a serial relationship established between the correlated or uncorrelated stimuli and the target (group Serial-Target and group Serial-Stimuli) would show evidence of differences in attention to the correlated and uncorrelated stimuli; and perhaps most interestingly whether these two groups would differ themselves. Finally, and in keeping with the design of Experiment 1, at the end of the Experiment 2, all groups received a questionnaire to determine their knowledge of the relationship between the correlated, uncorrelated, and target stimuli.

### Method

#### Participants

Fifty-two participants were recruited from the University of Nottingham’s School of Psychology and randomly assigned to either group Simultaneous, group Serial-Stimuli or group Serial-Target. Group Simultaneous consisted of 18 females with a mean age of 18.5 ± .86 (*M* ± *SD*) years; group Serial-Target consisted of 18 females with a mean age of 18.33 ± .49 years and group Serial-Stimuli consisted of 16 females with a mean age of 19 ± 1.27 years. Exclusion criteria for all groups were the same as in Experiment 1, and like in Experiment 1, all participants had normal or corrected-to-normal vision and received course credit for their participation or a £3 inconvenience allowance.

#### Apparatus and Stimuli

The apparatus used in Experiment 2 was identical to that used in Experiment 1. Stimuli consisted of the letters J, P, Q, V, W, and Z, which are better matched in terms of frequency in the English language than the letters used in Experiment 1. In keeping with Experiment 1, letters were presented in black, font size 60, Times New Roman. Letters Q and P always served as the target stimuli, while letters J, V, W, and Z were randomly assigned to serve as either the correlated or uncorrelated stimuli for each participant. For group Simultaneous the target appeared simultaneously with the correlated and uncorrelated stimuli at a random apex of the notional triangle described in Experient 1. For group Serial-Target and Serial-Stimuli, the target appeared alone at a random location within the notional triangle on the subsequent screen after the presentation of the compound of the correlated and uncorrelated stimuli.

Each of the four training trials was presented 36 times making 144 trials overall. Eight test trials were randomly distributed throughout the experiment in which four trials with each compound stimulus were presented twice in the absence of a target stimulus. On these trials, the correlated and uncorrelated stimuli were presented in the same manner as in Experiment 1.

#### Procedure

The same calibration, informed consent and health-screening procedure was used at the start of Experiment 2 as in Experiment 1. After this, participants read the following on-screen instructions: “*Each trial will begin with a fixation cross in the center of the screen. This will be followed by a series of letters. Your task is to indicate whether the letter ‘Q’ or ‘P’ is present in the array. Q = left arrow, P = right arrow. Pay attention to which stimuli are paired together because at the end you will have a memory test. Press any key to start the experiment.*” For group Serial-Stimuli, participants were additionally told, “*You can press early if you think you know which letter will appear*.” Each training trial began with the presentation of a fixation cross that was located in the center of the screen for 1,000 ms. This fixation cross was then removed and for group Simultaneous replaced with the triplet of the correlated, uncorrelated, and target letters, simultaneously, for 2,000 ms. For groups Serial-Stimuli and Serial-Target, the fixation cross was replaced with the presentation of the correlated and uncorrelated stimuli for 2,000 ms, which were then removed from the screen and followed immediately by the target stimulus, also for 2,000 ms. For all groups the trial then recycled, after a 1,000 ms blank screen, to the fixation cross. Although participants were told that they *could* press early if they thought the letter will appear in group Serial–Stimuli, they were free to respond at any point during the trial and could respond during the target is they wished. For each group, the stimuli remained on screen, irrespective of the presence of absence of responding, until the termination of the trial.

After participants completed this stage of the experiment, they were given a questionnaire to complete which tested their understanding of the relationship between the correlated and uncorrelated stimuli with target stimuli. With the exception of the identities of the letters used in Experiment 2, the questionnaire was the same as that used in Experiment 1.

### Results

The exclusion criteria were identical to those used in Experiment 1. Again zero participants were excluded.

#### Behavioral Data

**Figure 2 fig2:**
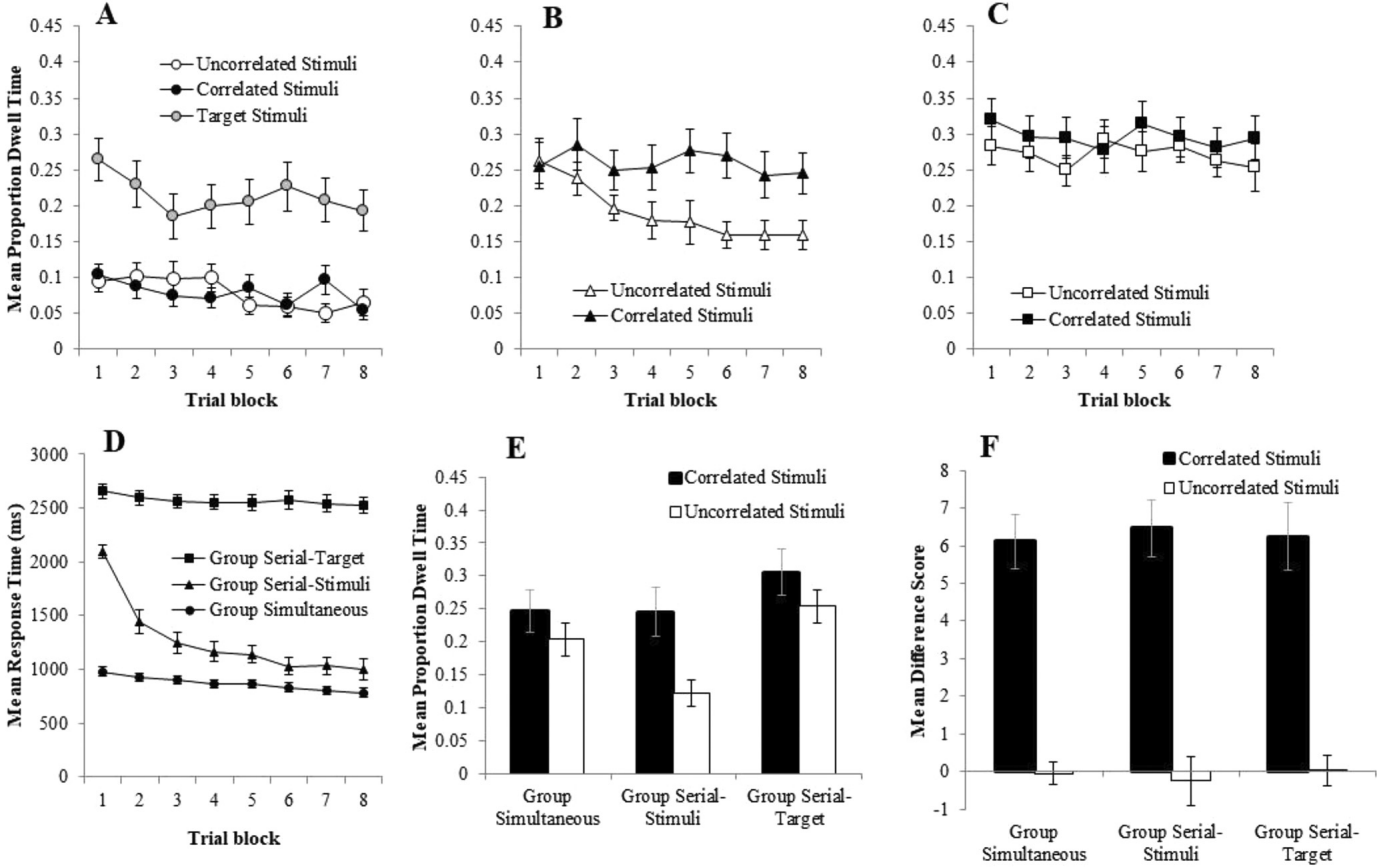
Results of Experiment 2 *Note.* (A) Mean proportion dwell times to the Correlated, Uncorrelated, and Target stimuli during the training trials in group Simultaneous; (B) mean proportion dwell times to the Correlated and Uncorrelated stimuli during the training trials in group Serial-Stimuli; (C) mean proportion dwell times to the Correlated and Uncorrelated stimuli during the training trials in group Serial-Target; (D) mean response times during the training trials in groups Serial-Target, Serial-Stimuli, and Simultaneous; (E) mean proportion dwell times to the Correlated and Uncorrelated stimuli during the probe trials in each group; and (F) mean difference scores to the Correlated and Uncorrelated stimuli during the final test questionnaire. Error bars represent 1 ± *SEM*.

In keeping with the results of Experiment 1, participants responded with high accuracy from trial-Block 1 onward, in all groups. In groups Simultaneous, Serial-Target, and Serial-Stimuli the mean proportions of correct responses during the final trial block were .98, .95, and .89, respectively. One sample *t* tests revealed that each of these means was significantly greater than chance (.5), smallest *t*(17) = 773.98, *p* < .001. The mean RT was again measured from the termination of the fixation cross and, as can be seen in Figure 2, Panel D, remained relatively constant for group Serial Target (Overall *M*: 2570 ms) and group Simultaneous (Overall *M*: 872 ms), and were consistent with participants in these groups responding at the point at which the target stimulus was presented. For participants in group Serial Stimuli, however, RTs during the first block of trials had a mean of around 2000 ms, before dropping quickly, and then terminating in the final block of training at a mean of around 1,000 ms. Thus, with training participants in this group shifted their response from the time in the trials when just the target was presented to a time in the trials when just the correlated and uncorrelated stimuli were presented. That is to say, they had acquired a predictive response. A two-way mixed ANOVA of mean RT with the within factor of trial block (1–8) and the between-subjects factor of group (Group Simultaneous vs. Group Serial-Stimuli vs. Group Serial-Target), revealed a main effect of group, *F*(2, 49) = 250.64, η*_p_*^2^ = .91, *p* < .001, of trial block, *F*(7, 343) = 17.85, η*_p_*^2^ = .27, *p* < .001, and an interaction between these factors, *F*(14, 343) = 7.32, η*_p_*^2^ = .23, *p* < .001. Bonferroni adjusted post hoc tests revealed that each group differed from each other group on each block (smallest mean difference = 305 ms, *SE* = 95.88, *p* = .008).[Fig fig2]

#### Eye Gaze Analysis

Panel A of Figure 2 show that there was no difference in the mean proportion of dwell time directed toward the correlated and uncorrelated stimuli on the training trials in group Simultaneous—a result that reproduces the effect observed in Experiment 1. Panel B of Figure 2 shows the proportion of dwell time for group Serial-Stimuli. For this group, a clear bias established as training progressed, with longer dwell time being spent toward the correlated stimuli relative to the uncorrelated stimuli. Finally panel C of Figure 2 shows the dwell time to the correlated and uncorrelated stimuli in group Serial-Target was comparable. A three-way ANOVA of mean proportions of dwell time with the within-subjects factors of stimulus type (correlated vs. uncorrelated), trial block (1–8), and the between-subjects factor of group (Group Simultaneous vs. Group Serial-Stimuli vs. Group Serial-Target) confirmed these impressions. This revealed a significant main effect of group, *F*(2, 26) = 27.84, η*_p_*^2^ = .68, *p* < .001, a main effect of trial block, *F*(3, 42) = 3.18, η*_p_*^2^ = .20, *p* = .031, but no main effect of stimulus type, *F*(1, 13) = 3.47, η*_p_*^2^ = .21, *p* = .085. There was, however, a significant three-way interaction between these factors, *F*(14, 182) = 1.83, η*_p_*^2^ = .12, *p* = .037, but no other interactions were significant, *F*s < 2.58, *p*s > .095. To further investigate the interaction between stimulus type (correlated vs. uncorrelated) and trial block (1–8) within each group, three two-way repeated measures ANOVAs were conducted. The first of these, conducted on the data from group Simultaneous, revealed no effect of stimulus type, *F*(1, 17) = .00, η*_p_*^2^ = .00, *p =* 1.00, a significant effect of trial block, *F*(7, 119) = 8.95, η*_p_*^2^ = .35, *p* < .001, and a significant interaction, *F*(7, 119) = 1073.30, η*_p_*^2^ = .98, *p* < .001, Simple main effects analysis of the interaction revealed a significant effect of stimulus type on Blocks 4 and 7, smallest, *F*(1, 17) = 5.41, η*_p_*^2^ = .24, *p* = .033, with first uncorrelated and then correlated stimuli receiving longer dwell times. The same analysis conducted on the data from group Serial-Target revealed no main effect of stimulus type, *F*(1, 17) = 2.52, η*_p_*^2^ = .13, *p* = .131, trial block, *F*(7, 119) = .75, η*_p_*^2^ = .04, *p* = .631, and no interaction, *F*(7, 119) = .55, η*_p_*^2^ = .03, *p* = .798. The same analysis performed on the data from group Serial-Stimuli, however, revealed a significant effect of stimulus type, *F*(1, 15) = 5.83, η*_p_*^2^ = 28, *p* = .029, trial block, *F*(4, 57) = 2.17, η*_p_*^2^ = .13, *p* = .088, but no interaction, *F*(3, 52) = .68, η*_p_*^2^ = .04, *p* = .588.

Panel E of Figure 2 shows dwell times to the correlated and uncorrelated stimuli from the test trials. These results largely confirm the observations from the training trials, with dwell times being longer to the correlated stimuli than the uncorrelated stimuli in group Serial-Stimuli (*t*(17) = 3.73, *p* = .002), but not in group Simultaneous (*t*(17) = 1.30, *p* = .221) or group Serial-Target (*t*(17) = 1.50, *p* = .153). JZS Bayes factors for these three *t* values were 24.07, 1.99, and 1.59 respectfully. These Bayes factors are in favor of the alternative, null and null hypotheses, again respectively.

#### Questionnaire Data

Panel F of Figure 2 shows that the mean difference score for the correlated stimuli was higher than that for the uncorrelated stimuli in all three groups. A two-way ANOVA of mean difference rating with the within-subjects factor of stimulus type (correlated vs. uncorrelated), and the between-subjects factor of group (Group Simultaneous vs. Group Serial-Stimuli vs. Group Serial-Target), revealed a main effect of stimulus type, *F*(1, 35) = 238.99, η*_p_*^2^ = .87, *p* < .001, no effect of group, *F*(2, 70) = .02, η*_p_*^2^ = .001, *p* = .978, and no interaction, *F*(2, 70) = .18, η*_p_*^2^ = .005, *p* = .835.

### Discussion

Experiment 2 reproduced the effect observed in Experiment 1 by demonstrating, again, that an attentional bias did not develop toward stimuli that were positively correlated with a copresent target event, even when participants had clearly learned the associative relationships between these events (group Simultaneous). The same outcome was also observed when a serial, rather than a simultaneous relationship was arranged between the correlated or uncorrelated stimuli and the target (group Serial-Target). An overt attentional bias was *only* established toward stimuli that were correlated with a target when participants were told that they could make a predictive response about the identity of the target, *before* its presentation (group Serial-Stimuli). Together, these results are difficult to reconcile with simple attentional models of associative learning that use only associations or associative error to determine attentional biases to stimuli that are correlated with a task-relevant events (e.g., Esber & Haselgrove, 2011; Le Pelley et al., 2011; Mackintosh, 1975). What these results suggest, instead, is that the prediction of a subsequent event is a key component for the allocation of learned attentional biases.

## Experiment 3

It is possible that the training given to group Serial-Target and group Simultaneous in Experiment 2 was, in fact, sufficient to bias attention toward the correlated stimuli; but, for some reason, the conditions of stimulus training (or exposure) may not have been appropriate to reveal this bias and masked its expression. In other words, the conditions of training used to vary attention, between groups, were confounded with the conditions of testing used to detect that bias. Part of this concern is alleviated by the test trials in Experiment 2 in which both groups were exposed to the correlated and uncorrelated stimuli in the absence of the target. However, on these trials an attentional bias toward the correlated stimulus may have been masked, or interrupted, by a search for the (now absent) target. To explore this possibility, in Stage 1 of Experiment 3, participants first received identical training to that given to participants in group Simultaneous or group Serial-Target in Experiment 2. On the basis of the results observed in Experiment 2 we expect to observe no difference in the dwell times toward the correlated and uncorrelated stimuli in this stage of the experiment. To determine if this training did, however, establish attentional biases to the correlated and uncorrelated stimuli that were, in some way, masked by the conditions of testing, all participants transferred to a second stage of the experiment in which the conditions of stimulus exposure and response instructions were the same as those given to group Serial-Stimuli in Experiment 2. Thus, in Stage 2, all participants were tested under the same circumstances and also under circumstances known (from Experiment 2) to permit the detection of a learned bias in overt attention.

A crucial manipulation in Stage 2 permitted us to evaluate whether any attentional bias established to the correlated cues in Stage 1 was unexpressed. For half of the participants within each of the two groups, the stimuli that were correlated with the target in Stage 1 remained correlated with the target in Stage 2 (and similarly the stimuli that were uncorrelated with the target in Stage 1 remained uncorrelated with the target in Stage 2)—generating groups Serial Congruent and Simultaneous Congruent. For the remaining participants within the Serial and Simultaneous groups, the stimuli that were correlated with the target in Stage 1 became uncorrelated with the target in Stage 2 (and similarly, the stimuli that were uncorrelated with the target in Stage 1 became correlated with the target in Stage 2)—generating groups Serial Incongruent and Simultaneous Incongruent (see Table 3). The logic behind this manipulation is that if attention was biased toward the correlated stimuli in Stage 1, but unexpressed, then in Stage 2 this bias should be revealed. This should particularly be the case in the two conditions where the contingencies between the stimuli and the targets remained the same between the two stages (that is to say there would be a behavioral saving in groups Serial Congruent and Simultaneous Congruent) relative to the two groups for whom the contingencies between the stimuli and the targets were reversed between Stages 1 and 2. For groups Serial Incongruent and Simultaneous Incongruent, the prior (putatively masked) biases, should hinder the expression of the attentional bias in Stage 2.[Table tbl3]

### Method

#### Participants

Each group consisted of 18 participants who were recruited from the University of Nottingham’s School of Psychology and randomly assigned to one of the four experimental groups. Group Serial-Congruent consisted of 11 females (*M ± SD* age: 19.90 ± 1.51); group Serial-Incongruent consisted of 12 females (19.83 ± 1.64); group Simultaneous-Congruent consisted of 12 females (21.08 ± 2.11); and finally group Simultaneous-Incongruent consisted of 12 females (20.67 ± 3.23). Exclusion criteria for all groups were the same as in Experiment 1, and like in Experiment 1, all participants had normal or corrected-to-normal vision and received course credit for their participation or a £3 inconvenience allowance.

#### Apparatus and Stimuli

The apparatus and stimuli used in Experiment 3 were identical to that used in Experiment 2. In Stage 1, each of the four trial-types was presented 18 times each in a random order. The spatial and temporal arrangement of the stimuli and trials for groups Serial-Congruent and Serial-Incongruent was identical to that given to group Serial-Target in Experiment 2. The spatial and temporal arrangement of the stimuli and trials for groups Simultaneous-Congruent and Simultaneous-Incongruent was identical to that in group Simultaneous in Experiment 2. There were no probe trials, in which the target was omitted in this stage of the experiment.

In Stage 2, the spatial and temporal arrangement of the stimuli and trials was the same for all groups, and identical to that given to group Serial-Stimuli in Experiment 2. At the end of Stage 2 for each group, the four trial types were presented six times in a random order in the absence of a target stimulus, and participants were asked to report which target stimulus they thought accompanied the compound during training.

#### Procedure

The eye tracker was calibrated and eye movement data were recorded in the same manner as described in Experiment 2. After performing the calibration, participants read on-screen instructions for Stage 1. For participants in all four groups, the Stage 1 instructions read: “*Each trial will begin with a fixation cross in the center of the screen. This will be followed by a series of letters. Your task is to indicate whether the letter ‘Q’ or ‘P’ is present in the array. Q = left arrow, P = right arrow. Pay attention to which stimuli are paired together because at the end you will have a memory test. Press any key to start the experiment.*”

Each trial during Stage 1 began with the presentation of a fixation cross in the center of the screen for 1,000 ms. After this, for groups Simultaneous Congruent and Simultaneous Incongruent, participants were presented with a triplet of the correlated, uncorrelated, and target stimuli for 2,000 ms. For groups Serial Congruent and Serial Incongruent the correlated and uncorrelated stimuli were presented for 2,000 ms followed by the target stimulus for 2,000 ms. For all groups, these sequences were followed by an interstimulus interval of 1,000 ms before the trial recycled with the fixation cross on the next trial.

Upon completing Stage 1, participants were presented with the following instructions: “*In the next stage you will see two letters followed by a target letter, but in addition to indicating its identity, this time you can anticipate whether the target will be a ‘P’ or a ‘Q’ by pressing early. Press the space bar to continue*.”

Each trial in Stage 2 began with the presentation of a fixation cross in the center of the screen for 1,000 ms. Participants were then presented with the correlated and uncorrelated stimuli for 2,000 ms, this compound was then followed by the target stimulus for 2,000 ms, this was followed by an interstimulus interval of 1,000 ms before the trial recycled to fixation cross for the next trial. Upon completing Stage 2, participants received a final series of test trials: “*In the final stage of the experiment you will be presented with two letters but they will not be followed by a target letter. Based on these you need to guess which letter should have been paired with them. Press the space bar to continue.*” In keeping with Experiment 2, for each group, the stimuli remained on screen irrespective of the presence of absence of responding, until the termination of the trial.

After participants completed this stage of the experiment, they were given a questionnaire to complete which tested their understanding of the relationship between the correlated and uncorrelated stimuli with target stimuli. The questionnaire was the same as that used in Experiment 2.

### Results

Exclusion criteria were identical to those used in Experiment 1, and again zero participants were excluded.

#### Stage 1

##### Behavioral Data

Participants in groups Simultaneous Congruent and Simultaneous Incongruent were treated identically in Stage 1, as were groups Serial Incongruent and Serial Congruent. The data for these two pairs of groups were combined for the analysis in Stage 1. During Stage 1 the mean proportion of correct responses was high (>.9) from Block 1, and increased only slightly over the course of the experiment. A two-way mixed measures ANOVA of individual proportions of correct responses with the within factor of trial block (1–4) and between factor of group (Simultaneous vs. Serial), revealed a significant main effect of trial block, *F*(3, 177) = 3.16, η*_p_*^2^ = .04, *p* = .034, and group, *F*(1, 70) = 6.47, η*_p_*^2^ = .09, *p* = .013, with the mean proportion correct being higher in the serial groups than in the Simultaneous groups. The interaction between block and group was not significant, *F*(3, 177) = 2.53, η*_p_*^2^ = .01, *p* = .657. The mean RT decreased slightly for the simultaneous and serial groups as training progressed. A two-way mixed measures ANOVA of individual RT with the within factor of trial block (1–4) and between factor of group (Simultaneous vs. Serial), revealed a significant main effect of trial block, *F*(2, 170) = 12.42, η*_p_*^2^ = .15, *p* < .001, and group, with RTs being faster in the simultaneous group, *F*(1, 70) = 4.61, η*_p_*^2^ = 4.61, *p* = .035. The interaction between block and group was not significant, *F*(2, 170) = 2.73, η*_p_*^2^ = .04, *p* = .057,

##### Eye Gaze Analysis

**Figure 3 fig3:**
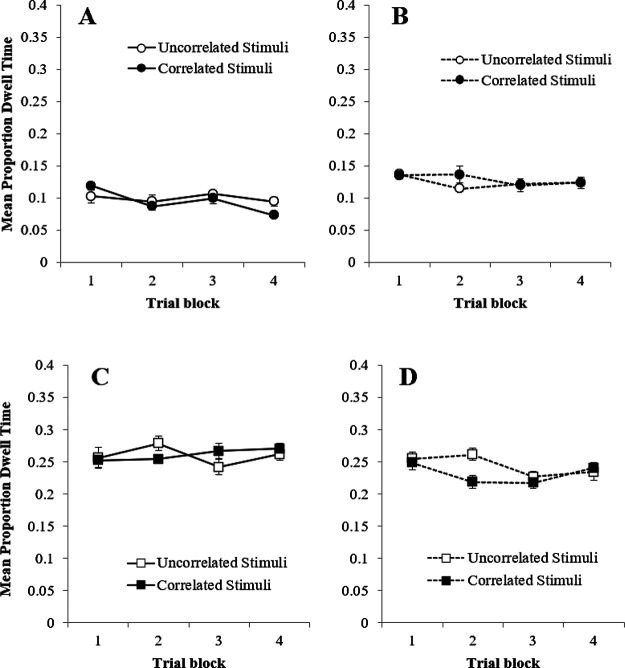
Results of Experiment 3 *Note.* Mean proportions of dwell times to the correlated and uncorrelated stimuli during the training trials of Stage 1 of Experiment 3 in groups (A) Simultaneous Congruent, (B) Simultaneous Incongruent, (C) Serial Congruent, and (D) Serial Incongruent. In each panel, the correlated or uncorrelated status refers to the relationship between the stimuli and the target during Stage 1. Error bars represent 1 ± *SEM*.

In keeping with the outcome of Experiment 2, Panels A, B, C, and D of Figure 3 demonstrates that there was no difference in the mean proportion of dwell time directed toward the correlated and uncorrelated stimuli for any of the groups over the trial blocks of Stage 1. A four-way mixed measures ANOVA of mean proportion of dwell time with the within factors of trial block (1–4) and stimulus type (correlated vs. uncorrelated), and between factors of group (Simultaneous vs. Serial) and congruency (Congruent vs. Incongruent) revealed a significant main effect of group, *F*(1, 34) = 68.16, η*_p_*^2^ = .67, *p* < .001, all remaining main effects and interactions were nonsignificant (largest *F*(1, 34*)* = 2.23, η*_p_*^2^ = .06, *p* = .144).[Fig fig3]

#### Stage 2

##### Behavioral Data

**Figure 4 fig4:**
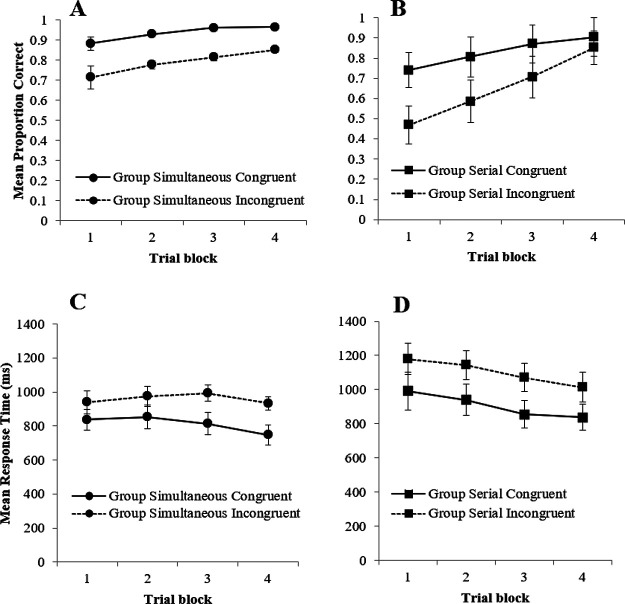
Results of Experiment 3 *Note.* Panels A and B: Mean proportion correct responses during Stage 2 of Experiment 3—(A) groups Simultaneous Congruent and Incongruent, (B) groups Serial Congruent and Incongruent. Panels C and D: Mean response times during Stage 2 of Experiment 3—(C) groups Simultaneous Congruent and Incongruent, (D) groups serial Congruent and Incongruent. Error bars represent 1 ± *SEM*.

Panels A and B of Figure 4 show the mean proportion of correct responses for the four groups during Stage 2. Performance improved across the four blocks of training and, across the majority of these blocks, accuracy was superior in the congruent groups, regardless of whether training involved serial or simultaneous training in Stage 1. A three-way mixed measures ANOVA of mean proportion correct responses with the within-group factor of trial block (1–4), and between factors of group (Simultaneous vs. Serial) and congruency (Congruent vs. Incongruent), revealed a significant main effects of trial block, congruency and group, smallest *F*(1, 31) = 6.42, η*_p_*^2^ = .17, *p* = .017. There was a significant Block × Group interaction, *F*(3, 93) = 3.58, η*_p_*^2^ = .10, *p* = .017, and Block × Congruency interaction, *F*(2.41, 74.57) = 4.39, η*_p_*^2^ = .12, *p* = .011. However, the interaction between congruency and group, and the three-way interaction were not significant, largest *F*(3, 93) = 1.66, η*_p_*^2^ = .05, *p* = .182. Simple main effects analysis of the interaction between block and group revealed that the difference in proportion of correct responses between group Simultaneous and group Serial was significant in Block 1 and 2, smallest, *F*(3, 93) = 6.90, η*_p_*^2^ = .33, *p* = .020 with group Simultaneous having a higher proportion of correct responses. Simple main effects analysis of the interaction between block and congruency revealed that the difference in proportion of correct responses for group Congruent and group Incongruent was significant in Block 1, 2, and 3 smallest, *F*(2.41, 74.57) = 11.01, η*_p_*^2^ = .44, *p* = .005 with group Congruent having a higher proportion of correct responses. This final interaction, as well as the main effect of congruency confirms that our manipulation of congruency between Stage 1 and 2 had a detectable impact upon behavior—presumably because of a direct transfer of associative strength from the stimuli that remained correlated with the target in Stage 1 and 2 in the two congruent groups.[Fig fig4]

Panels C and D of Figure 4 show that in Stage 2, the mean RT decreased across training in both the simultaneous and serial groups, but was fastest for the congruent conditions, again confirming that switching the contingency of the correlated and uncorrelated stimuli between experimental stages was effective, and that participants were sensitive to the difference in correlation of the stimuli during Stage 1 of the Experiment. A three-way mixed measures ANOVA of mean RT with the within factor of trial block (1–4), and between factors of group (Simultaneous vs. Serial) and congruency (Congruent vs. Incongruent) revealed significant main effects of trial block, group, and congruency, smallest *F*(2.01, 58.19) = 4.30, η*_p_*^2^ = .13, *p* = .018, and a significant Block × Group interaction, *F*(3, 87) = 4.30, η*_p_*^2^ = .13, *p* = .007. All remaining interactions were nonsignificant, largest *F*(1, 29) = 1.26, η*_p_*^2^ = .04, *p* = .270.

##### Eye Gaze

**Figure 5 fig5:**
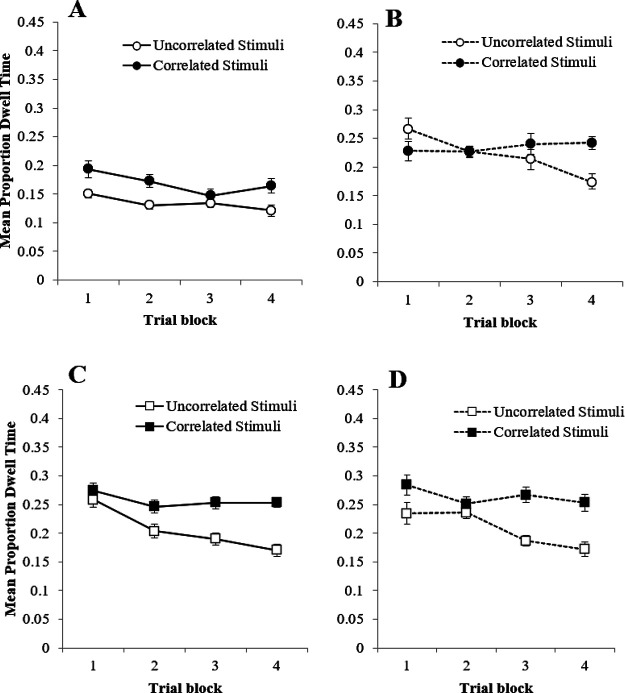
Results of Experiment 3 *Note.* Mean proportion dwell times to the correlated and uncorrelated stimuli during Stage 2 of Experiment 3. (A) group Simultaneous Congruent (B), group Simultaneous Incongruent (C), group Serial Congruent, and (D) group Serial Incongruent. In each panel, the correlated or uncorrelated status refers to the relationship between the stimuli and the target during Stage 2. Error bars represent 1 ± *SEM*.

Panels A, B, C, and D of Figure 5 show that a larger proportion of dwell time was directed toward the correlated stimuli in all groups in Stage 2, irrespective of whether participants were in the congruent or incongruent groups, or whether training in Stage 1 was conducted using a serial or simultaneous procedure. A four-way mixed measures ANOVA of mean proportion of dwell time with the within factors of trial block (1–4), stimulus type (correlated vs. uncorrelated), and between-group factors of group (Simultaneous vs. Serial) and congruency (Congruent vs. Incongruent) revealed significant main effects of block, stimulus type, and group, smallest *F*(1, 34) = 4.62, η*_p_*^2^ = .12, *p* = .039, the remaining main effect of congruency was not significant, *F*(1, 34) = 3.30, η*_p_*^2^ = .09, *p* = .078. The scaled JZS Bayes factor for this effect was 1.038, in favor of the null. There was a significant Block × Stimulus Type interaction, *F*(3, 102) = 3.19, η*_p_*^2^ = .09, *p* = .027, but all remaining interactions were nonsignificant, largest *F*(1, 34) = 2.61, η*_p_*^2^ = .07, *p* = .116.[Fig fig5]

**Figure 6 fig6:**
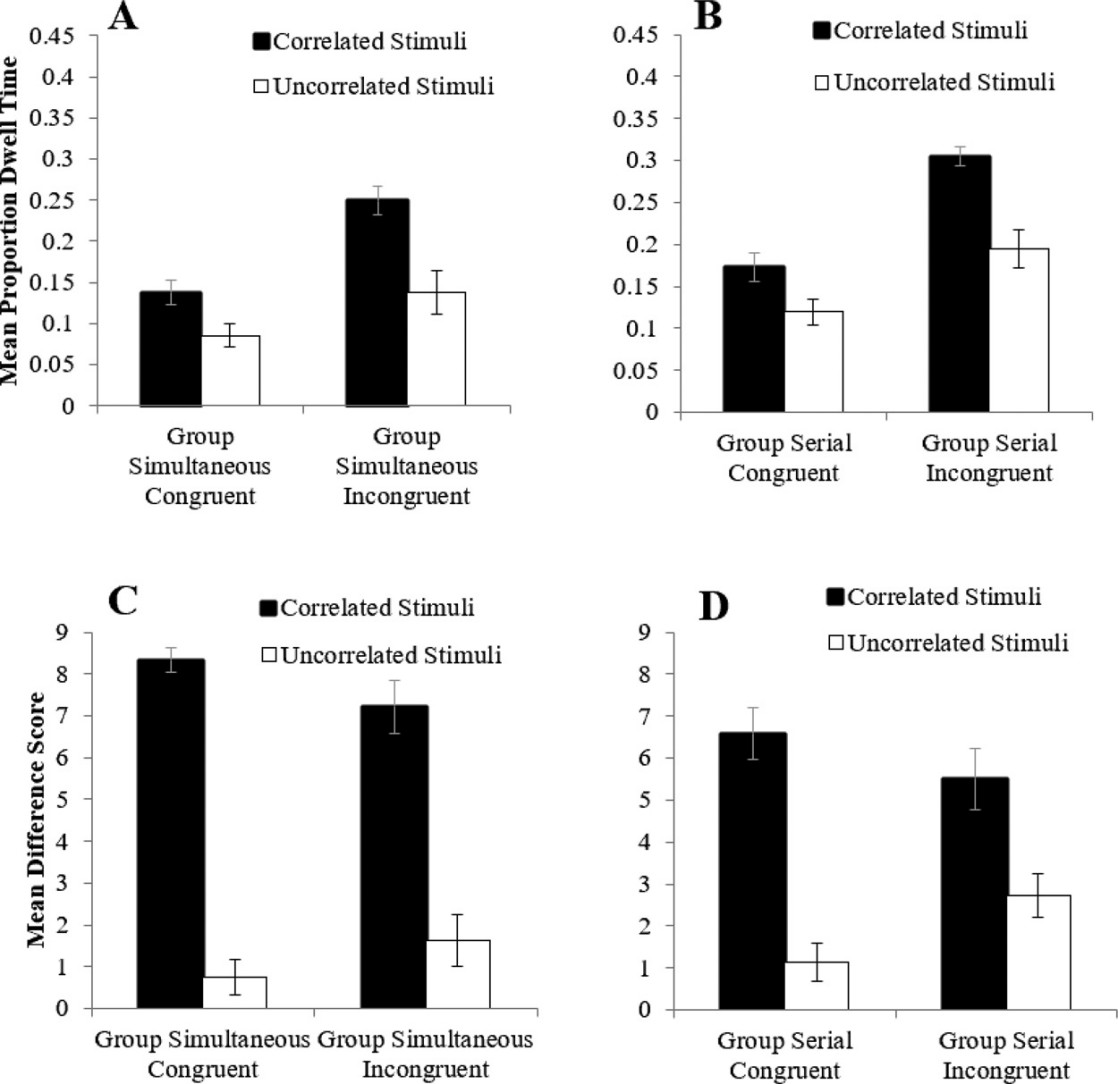
Results of Experiment 3 *Note.* Mean proportion dwell times to the correlated and uncorrelated stimuli during probe trials in groups Simultaneous Congruent and Simultaneous Incongruent (A) and groups Serial Congruent and Serial Incongruent (B). Mean difference scores to the correlated and uncorrelated stimuli from the final questionnaire in groups Simultaneous Congruent and Simultaneous Incongruent (C) and groups Serial Congruent and Serial Incongruent (D). Error bars represent 1 ± *SEM*.

Panels A and B of Figure 6 show the mean proportion of dwell times directed to the correlated and uncorrelated stimuli during the test trials for all groups in Stage 2. Overall, dwell times in the incongruent groups were longer than in the congruent groups, perhaps reflecting the uncertainty associated with the introduction of the manipulation Stage 2 (Pearce & Hall, 1980). In keeping with the data from the training trials, correlated stimuli attracted the largest proportion of dwell time in groups trained simultaneously or serially in Stage 1. A three-way repeated measures ANOVA of mean proportion of dwell time with the within-subjects factor of stimulus type (correlated vs. uncorrelated) and between-groups factors of group (Simultaneous vs. Serial) and congruency (Congruent vs. Incongruent) revealed a significant main effect of stimulus type, *F*(1, 68) = 22.77, η*_p_*^2^ = .25, *p* < .001, group, *F*(1, 68) = 4.71, η*_p_*^2^ = .07, *p* = .034, and congruency, *F*(1, 68) = 14.19, η*_p_*^2^ = .17, *p* < .001. However, all interactions were nonsignificant, largest, *F*(1, 68) = 2.64, η*_p_*^2^ = .04, *p* = .109.[Fig fig6]

##### Questionnaire Data

Difference scores for all four groups were calculated in a manner identical to Experiment 2 and are shown in panels C and D of Figure 6. A three-way repeated measures ANOVA of mean difference scores with the within factor of stimulus type (correlated vs. uncorrelated) and between factors of group (Simultaneous vs. Serial) and congruency (Congruent vs. Incongruent) revealed a significant main effect of stimulus type, *F*(1, 68) = 149.83, η*_p_*^2^ = .688, *p* < .001, but no effect of group or congruency, largest *F*(1, 68) = 2.12, η*_p_*^2^ = .03, *p* = .145. Significant interactions were found between Stimulus Type × Congruency (*F*(1, 68) = 7.13, η*_p_*^2^ = .10, *p* = .009), Stimulus Type × Group (*F*(1, 68) = 8.01, η*_p_*^2^ = .11, *p* = .006), but the three-way interaction was nonsignificant (*F*(1, 68) = .146, η*_p_*^2^ = .002, *p* = .704). Simple main effects analysis of the interaction between stimulus type and congruency revealed that the difference in difference rating between correlated and uncorrelated stimuli was significant in congruent and incongruent groups, smallest, *p* < .001. Simple main effects analysis of the interaction between stimulus type and group revealed that the difference in difference rating between correlated and uncorrelated stimuli was significant in the simultaneous and serial conditions, smallest, *p* < .001. Thus, in keeping with the previous experiments, participants knowledge of the associative relations between the correlated or uncorrelated stimuli and the target was good.

### Discussion

The eye-tracking data from Stage 2 of the current experiment, reproduced the effect observed in group Serial-Stimuli from Experiment 2: when participants were required to make a predictive response about the identity of a subsequent task-relevant target then their overt attention came to be biased toward the stimuli correlated with the target. The data from Stage 1 reproduce the effects observed in groups Serial-Target and Simultaneous from Experiment 2: when a simultaneous or sequential relationship between correlated stimuli and target is established—without the requirement of predictive response—then there was no indication of the acquisition of an attentional bias. Of most interest, the current experiment revealed that when the predictive contingencies of the correlated and uncorrelated stimuli were swapped between Stage 1 and 2 then there was no disruption in the bias in overt attention to the correlated stimuli in Stage 2. In fact, numerically, the difference in dwell times between the correlated and uncorrelated stimuli was most substantial in the Incongruent groups than in the Congruent groups. Thus, it does not appear that the simultaneous and sequential training in Stage 1 established an attentional bias that, for whatever reason, went undetected. If this were the case then we would anticipate seeing an attenuation of the difference in dwell time between the correlated and uncorrelated stimuli in the Incongruent groups in Stage 2, which was not observed. It is worth reiterating that the behavioral data (mean proportion correct and RTs) and the overall dwell times were different between the incongruent groups and the congruent groups in Stage 2; thus, providing a confirmation of the effectiveness of this manipulation. Finally, all groups showed appropriate knowledge about the associative relationships between the correlated stimuli and the targets. The results of the current experiment, together with Experiments 1 and 2, suggest that mere association or associative error is insufficient to result in learned changes in overt attention.

## General Discussion

The purpose of the experiments reported here was to investigate the role of prediction in learned predictiveness. Prior studies of this phenomenon (e.g., Le Pelley et al., 2011) have revealed that a stimulus correlated with a task-relevant event comes to control more overt visual attention than a stimulus that is task irrelevant. These studies have been taken to support the “predictiveness principle”: the idea that, through learning, stimuli that are “predictive” of events of importance come to control relatively more attention. However, as we have noted, formal models that are used to simulate these effects (e.g., Esber & Haselgrove, 2011; Le Pelley, 2004; Mackintosh, 1975) use association and associative error to bridge the relationship between learning and attention, and make no reference to time. Consequently, these models make no explicit dissociation between sequentially and simultaneously presented events, and are silent with respect to the actual role of prediction in learned predictiveness.

Experiment 1 revealed that despite participants’ having appropriate associative knowledge about the relationship between stimuli that were correlated or uncorrelated with a target stimulus, no bias in visual dwell time was observed toward the correlated stimulus when all these stimuli were presented simultaneously. This result implies that mere association between stimuli is insufficient to modify learned variations in attention. Experiment 2 reproduced this effect in group Simultaneous, and also revealed that arranging for the correlated and uncorrelated stimuli to precede the target stimulus was, in and of itself, also insufficient to result in the acquisition of a bias in visual dwell time to the correlated stimuli (group Serial-Target). This was again, in the presence of appropriate associative knowledge about the relationship between these stimuli and the target. Thus, a veridical, predictive, relationship between *stimuli* is insufficient for learning to modify attention. Only when participants were asked to make a predictive *response*, that is to say a response before the presentation of the target, were longer dwell times acquired to the correlated stimulus relative to the uncorrelated stimulus. Experiment 3 confirmed these findings, and also provided evidence that the lack of attentional biases in groups Simultaneous and Serial-Target were not a consequence of a confound between the conditions of training and the conditions of testing.

**Figure 7 fig7:**
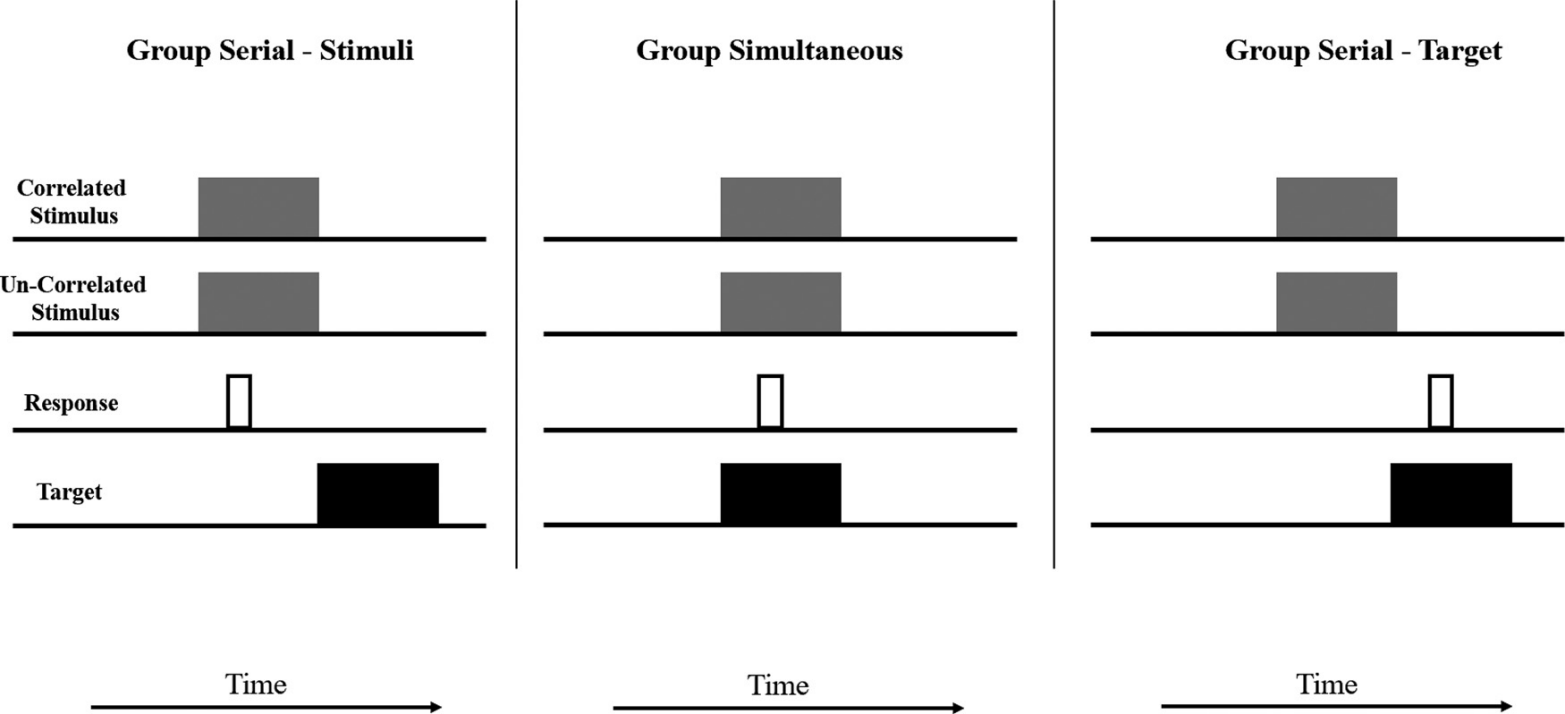
A Time Line Depicting the Relative Presentation Times of the Correlated and Uncorrelated Stimuli (Grey Rectangles) Participant’s Response (White Rectangle) and the Target (Black Rectangle) During a Trial in Groups Serial-Stimuli, Simultaneous, and Serial-Target Of Experiment 2

Together, these results imply a crucial role for predictive responding in the etiology of the learned predictiveness effect. More specifically they suggest that responding needs to be contingent with a stimulus at a time when the target is not present for learning to change overt visual attention. To make this clear, consider Figure 7, which shows a timeline of one trial for group Serial-Stimuli, group Simultaneous, and group Serial-Target from a relatively late part of training in Experiment 2. Note that the temporal relationships between the correlated stimulus and the Target stimulus was equivalent in group Serial-Stimuli and group Serial-Target. Thus, the contiguity between these events *alone* was not sufficient to explain the bias in visual attention evident in group Serial-Stimuli. Furthermore, relative to the onsets of the correlated and uncorrelated stimuli, responses are produced at comparable times in groups Serial-Stimuli and group Simultaneous. Consequently, the contiguity between the correlated stimulus and the response *alone* is not sufficient to explain the bias in visual attention evident in group Serial-Stimuli but not in group Simultaneous. To explain the attentional bias toward the correlated stimulus observed in group Serial-Stimuli, but not in the other groups, we postulate that the target-relevant response must be performed contiguously with the correlated stimulus, *at a time when the target is not present*.[Fig fig7]

It is relatively straightforward to, algorithmically, modify the equations provided by associative models of learning to realize the conceptual description provided above. For example, taking Mackintosh’s theory as a case in point, we could stipulate that Equations 2a and 2b described in the beginning of the article only take effect when the task-relevant response occurs at a time before the onset of the task-relevant target (i.e., t_CR_ < t_λ_). In the context of Mackintosh’s theory this would mean that associative learning would still take place (as Equation 1 is not limited by the temporal relationship between response and target); however, this learning will not be translated into changes in attentional control, unless the task relevant responding preceded the presentation of the target stimulus. Consequently, this modification to Mackintosh’s theory would explain why all three groups in Experiment 2 demonstrated good, and equivalent, knowledge of the associative relationships between the correlated or uncorrelated stimuli and the target but why only group Serial-Stimuli translated this knowledge into a change in attention.

There are, however, some problems with this algorithmic fudge. The first is relatively minor as it applies only to the modification as it is applied to the Mackintosh (1975) model. Note that learning, in Equation 1, is driven with an individual error term (e.g., Bush & Mosteller, 1951), rather than the summed error term used more standardly (e.g., Rescorla & Wagner, 1972). The summed error term is only used, in Mackintosh’s theory, to change attention to stimuli in Equations 2a and 2b. Consequently, effects such as blocking (Kamin, 1968), or overshadowing (Pavlov, 1927) are driven only by changes in the allocation of attention to stimuli (specifically, a reduction in attention to redundant stimuli, Jones & Haselgrove, 2011, 2013). It follows then, that if we restrict attentional change to circumstances in which task-relevant responding precedes presentation of the target then overshadowing and blocking should not be evident under circumstances in which a conditioned, or target-relevant response, coincides with the unconditioned, or target, stimulus; a prediction that is demonstrably false (e.g., Balleine et al., 2005; Dwyer et al., 2011). Fortunately, this problem can be overcome if we relax the assumption that the summed error term is applied only to the mechanism that changes the allocation of attention. If a summed error term is applied at the level of the learning algorithm too (e.g., Esber & Haselgrove, 2011; Le Pelley, 2004), then more than one source of cue-competition is available and effects such as simultaneous-stimulus blocking and overshadowing can be explained, albeit in a manner that does not permit the stimuli used in these studies to undergo a change in attention.

**Figure 8 fig8:**
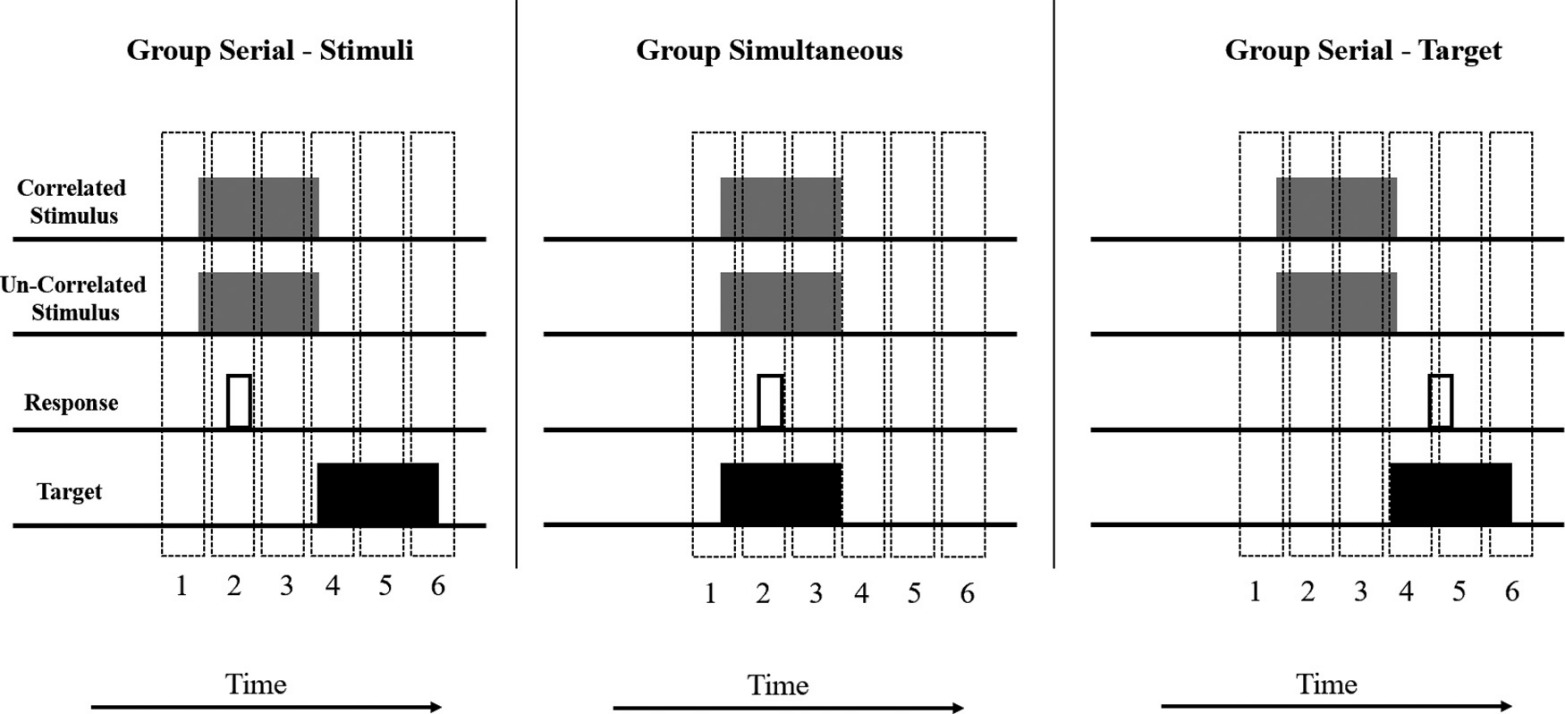
A Time Line Depicting the Relative Presentation Times of the Correlated and Uncorrelated Stimuli (Grey Rectangles) Participants Response (White Rectangle) and the Target (Black Rectangle) During a Trial in Groups Serial-Stimuli, Simultaneous, and Serial-Target of Experiment 2 *Note.* Dotted rectangles represent six theoretical windows of associability.

The second problem with the modification described above is more fundamental and applies to theories of learning and attention more generally. As we have seen, most models of attentional learning (e.g., Esber & Haselgrove, 2011; Le Pelley, 2004; Mackintosh, 1975), which can successfully explain a broad range of learning phenomena, do so without taking into account how time is represented within the architecture of the model—they are so called trial-based models. Consequently, before any such algorithmic modification may be applied, one must first begin to tackle the question of precisely how the timing of stimuli and their associated responses will be represented. A common approach to this problem is to acknowledge that rather than discrete trials being the smallest unit of temporal resolution within learning, instead, shorter windows of associability (epochs, or “bins”; e.g., Moore & Stickney, 1980) are assumed to successively open and close as time passes, with experimental events potentially spanning multiple epochs. Applying this assumption may provide a resolution of the experiments that we report here. Consider Figure 8, which for the sake of illustration and simplicity, redraws Figure 7 to exemplify how a single trial may be represented across six different windows of associability that open and close during this series of experimental events. We make the assumption that associations between any experimental event (stimuli or responses) will be more successfully acquired when those events occupy the same epoch (e.g., Moore & Stickney, 1980). We also make the assumption (that we will discuss later) that attention to stimuli is modified not on the basis of their association with other stimuli, but on the basis of their association with the task-relevant *response*.[Fig fig8]

On the basis of these assumptions, it can be seen that in each group there is one or more epoch in which the correlated stimulus and the target stimulus are both coactive (epoch 4 for the two Serial groups, and epochs 1 to 4 for group Simultaneous). Consequently, associative learning will have the opportunity to take place between these stimuli and a test of associative knowledge (such as the test trials presented at the end of each experiment) has the opportunity to reveal this. Similarly, it can also be seen that the correlated stimulus and the response are also both coactive (in epoch 2 for groups Serial-Stimuli and Simultaneous, and in epoch 4 for group Serial-Target). Note, however, that for groups Simultaneous and Serial-Target the correlated stimulus and the response are both coactive within the same epoch as the target stimulus. On the basis of most learning rules, therefore, the association between the correlated stimulus and the response will be overshadowed by the target stimulus. This will not be the case in group Serial-Stimuli, however, because for this group the target is not presented during an epoch in which the correlated stimulus and the response are coactive; consequently the influence of overshadowing by the target will be far less. If, as we have postulated above, attentional control by stimuli (in this case the correlated stimulus) is a function of its association with the task-relevant response, then what should be observed is the acquisition of an attentional bias in group Serial-Stimuli, but not in the remaining two groups, which is of course the result we observed. Our proposed mechanism for this is a combination of standard associative principles (in this case overshadowing) operating at the epoch level—a proposal which is not particularly controversial (McLaren & Mackintosh, 2000; Vogel et al., 2003) and an attentional modulation mechanism that is sensitive to the correlation of stimulus and task-relevant responding. It is to this second proposal that we now turn our attention.

While associative theories typically emphasize the role of attention to be one that permits organisms to better select stimuli on the basis of their predictive validity of other subsequent *stimuli* (e.g., an outcome), an alternative view of attention is afforded by a number of cognitive models, which instead emphasize the primary role of attention to be one of controlling action (e.g., Allport, 1989; Norman & Shallice, 1986). By placing the emphasis on the relationship between stimuli and responses, the proposals outlined here can be viewed as consistent with the idea that attention is bound to the intended actions of the participant (e.g., Engel et al., 2013). For example, according to premotor theories of attention, the selection of sensory information is determined by current action plans (Rizzolatti et al., 1987). Notably, Craighero et al. (1999) state the general position as one in which “Orienting of attention implies an activation of basic sensory-motor circuits according to the action goal. *Attention results, therefore, from an internal representation of the required response during the interval between cue presentation and target presentation*” (p. 1690). Similarly, so-called late-selection models of attention (e.g., Deutsch & Deutsch, 1963; Duncan, 1980; Kahneman, 1973) assume that attentional selection, or filtering, occurs at a relatively late stage in information processing (cf. Broadbent, 1958). According to models of this class, the selection of stimuli for attention involves more complex, “categorical” information than mere physical stimulus properties. As Deutsch and Deutsch note, “all sensory messages which impinge on the organism are perceptually analyzed at the highest level” (p. 85), and as Quinlan and Dyson (2008) note, in their discussion of late selection models more generally, “It is as if everything is identified but that *the critical constraints relate to responding*” (p. 286). The proposal we suggest here, in which learned changes in attention are a consequence of stimulus–response associations, concords with the idea that the purpose of attention is to guide actions. Perhaps this position has some face validity too. If the organism finds itself engaged in a task in which the constraints require the selection of information, then it may well be a consequence of the limits of processing capacity being either reached, or at least approached. Under these circumstances, then, a relatively automatic mechanism for determining how attention should be allocated seems necessary. A direct association between stimulus and response fulfils this requirement.

By proposing that learned variations in stimulus attention are a function of the strength of the association between that stimulus and the task-relevant response, we align the current results with a literature that suggests that attentional selection for reward associated stimuli is a consequence of an attentional habit (e.g., Anderson, 2016; Luque et al., 2017). According to this position, an attentional habit is conceptually similar to the architecture (stimulus-response) of a habit as considered in instrumental-learning paradigms. For example, lever-press responding for food comes under the control of a particular stimulus (e.g., the sight of the lever)—an association that is reinforced by reward (Dickinson, 1985). When applied to an attentional habit, the notion here is that eye movements can be thought of as the instrumental response that has come under the control of experimental stimuli (e.g., cues predictive of target outcomes). The associative structure of the proposals we describe here are in keeping with this general view; however, our suggestion is that the association between stimuli and task-relevant motor responses (such as key presses) in addition to eye movements, also modifies overt attention. A counterpoint to this possibility is worthy of note, however. In studies of value modulated attentional capture (e.g., Anderson et al., 2011a, 2011b) a stimulus that is established as predictive of a large monetary reward will come to attract more visual attention than a stimulus that is predictive of a smaller monetary reward; and importantly, this effect can even be observed when orienting responses to the stimulus associated with the high-value reward are detrimental to ongoing task-relevant performance (Le Pelley et al., 2016). It is difficult to see how a stimulus-response analysis, such as that developed here, could account for these results.

Our analysis, thus far, has focused on the role of differences in the timing of responding during stimuli in the etiology of learned predictiveness. However, it is important to consider that the three groups in Experiment 2, and the conditions of Stage 1 and Stage 2 in Experiment 3 also differed in terms of the instructions that participants were provided with, and that this difference is an equally good predictor of which conditions or groups demonstrated an attentional bias to the correlated stimuli. Specifically, when participants were instructed that they may anticipate the arrival of the target stimulus, and press the response key early, then only under these circumstances did dwell times come to be longer to the correlated stimuli relative to the uncorrelated stimuli. It is worthwhile considering whether variations in the instructions were responsible for the effects observed in the current studies. Evidence for the role of instruction in learned variations in attention has been provided by Mitchell et al. (2012). In their Experiment 2, participants were required to learn to predict the shape that a tree would grow into depending on the sets of cross-pollinating seeds that were used. Their results revealed a standard learned predictiveness effect, in that dwell times were longer to the stimuli (seeds) that were established as predictive of the outcome (tree shape) than to stimuli that were irrelevant to the task. However, this bias in dwell time could be reversed in a second stage of the experiment if participants were given instructions that informed them that it was highly unlikely that the seeds that controlled the shape of the tree previously would influence the shape of the trees from now on. Subsequent studies have also revealed that instructions can influence learned predictiveness; however, a residual bias toward previously predictive cues can survive the instructions (Don & Livesey, 2015; Shone et al., 2015). Mitchell et al., account for their results by suggesting that the learned predictiveness effect is a consequence of participants’ controlled attention being determined (in part) by a “causal model” of the scenario described in the cover story of the task used in their experiment, a model that can be revised on the basis of instruction (see also Mitchell et al., 2009). Consequently, it is clear that the nature of the instructions given to participants can have a substantial influence on how learned variations in overt visual attention are expressed to stimuli that are correlated or uncorrelated with task-relevant goals.

One possible way in which instructions may have impacted overt visual attention in the current studies is through motivating differing strategies. For example, in both group Simultaneous and group Serial-Target in Experiment 2, participants were not required to predict a subsequent event on each trial because their task instructions emphasized responding to the target stimulus when it is seen. On this basis, then, changes in attention may only take place when participants are required to predict a future event, as was the case in group Serial-Stimuli in Experiment 2. One way to dissociate this analysis from the analysis based upon S-R overshadowing presented earlier, would be to conduct a backward learned predictiveness study. Here participants would be presented with outcomes or targets *before* the presentation of compounds of correlated and uncorrelated stimuli and instructed to respond as to the identity of the target during the compound. According to the instructional analysis just developed, under these circumstances, we would expect no attentional bias to develop to the correlated stimuli as the instructional requirement is to make a response about the identity of a past event rather than a prediction about a future event. However, according to the S-R overshadowing analysis, an attentional bias to the correlated stimulus *should* still develop as the response is being made contiguously with the presentation of the correlated stimulus, but in the absence of the target stimulus, and hence the contribution of overshadowing of the correlated S-R association would be less.^2^

In any case, the current experiments reveal that in order for learning to modify attention to a stimulus that is correlated with a target, task-relevant responding must take place at a time before target presentation. Mere “association with some other immediately interesting thing” (James, 1890) is insufficient for a stimulus to derive attention. It seems, then, that learned predictiveness is appropriately named—but perhaps for not the reason suspected by associative theories of learning.

## Figures and Tables

**Table 1 tbl1:** Design of the Training Received by Participants in Le Pelley et al. (2011)

Outcome 1	Outcome 2
AV	BV
AW	BW
DX	CX
DY	CY
*Note*. Letters A to D, and V to Y denote nonsense words presented on screen. Outcomes 1 and 2 denote the sounds participants were required predict based upon the identity of the nonsense words.	

**Table 2 tbl2:** Example Design of Experiment 1

Training trials	Correct response (target)	Test trials
U W Y	Y	U W
U X Y	Y	U X
V W Z	Z	V X
V X Z	Z	V W
*Note*. Triplets of letters comprising correlated, uncorrelated, and target stimuli were presented on screen. Responses to the presentation of either of two target stimuli (e.g., Y and Z) were required. Test trials with the correlated and uncorrelated stimuli alone were interspersed throughout training with the training trials.		

**Table 3 tbl3:** Example Design of Experiment 3

Group	Stage 1 (Respond to Target)	Stage 2 (Predict Target)	Test trials
Simultaneous	**J**WQ	**J**W→Q	JW
Congruent	**J**ZQ	**J**Z→**Q**	JZ
	**V**WP	**V**W→P	VW
	**V**ZP	**V**Z→P	VZ
Simultaneous	**J**WQ	J**W**→**Q**	JW
Incongruent	**J**ZQ	J**Z**→P	JZ
	**V**WP	V**W**→Q	VW
	**V**ZP	V**Z**→P	VZ
Serial	**J**W→Q	**J**W→Q	JW
Congruent	**J**Z→Q	**J**Z→Q	JZ
	**V**W→P	**V**W→P	VW
	**V**Z→P	**V**Z→P	VZ
Serial	**J**W→Q	J**W**→Q	JW
Incongruent	**J**Z→Q	J**Z**→P	JZ
	**V**W→P	V**W**→Q	VW
	**V**Z→P	V**Z**→P	VZ
*Note*. Target stimuli are underlined. Stimuli correlated with the target, within a stage, are in bold.			

## Footnotes

1 The logic of the group nomenclature is as follows. *Simultaneous* and *Serial* refer to the nature of the presentation of the stimuli on each trial. When the correlated, uncorrelated and target stimuli are presented at the same time then the group designation is simultaneous; when the correlated and uncorrelated stimulus precede the target, the group designation is serial. *Stimuli* and *Target* distinguish the two serial groups with respect to the response instructions. When the instructions indicate responding is to the Target then the designation is *Target*; when the instructions also inform that responding may take place during the stimuli then the designation is *Stimuli.*

2 We acknowledge that this analysis ignores the potential for a trace representation of the target stimulus to overshadow the association between the correlated stimulus and the response.
